# Improvement of cool-season food legumes for adaptation to intercropping systems: breeding faba bean for intercropping with durum wheat as a case study

**DOI:** 10.3389/fpls.2024.1368509

**Published:** 2024-05-16

**Authors:** Lynn Abou Khater, Fouad Maalouf, Rind Balech, Yuhua He, Xuxiao Zong, Diego Rubiales, Shiv Kumar

**Affiliations:** ^1^ Biodiversity and Crop Improvement Program, International Center for Agricultural Research in the Dry Areas (ICARDA), Terbol, Lebanon; ^2^ Institute of Food Crop, Yunnan Academy of Agricultural Science, Kunming, China; ^3^ Institute of Crop Sciences, Chinese Academy of Agricultural Sciences, Beijing, China; ^4^ Institute for Sustainable Agriculture, The Spanish National Research Council (CSIC), Córdoba, Spain; ^5^ Biodiversity and Crop Improvement Program, International Center for Agricultural Research in Dry Areas (ICARDA), New Delhi, India

**Keywords:** cool season food legumes, intercropping, faba bean, breeding, traits for intercropping

## Abstract

Although the transition toward a more sustainable agricultural system is sparking the interest of scientists and farmers around the globe, breeding programs are still focusing on optimizing cultivars intended for the monoculture system, and most cultivars available on the market are not suitable for intercropping. The incorporation of versatile cool-season food legumes (CSFLs) in the intercropping system is a promising way toward more diversified and sustainable cropping systems. However, as the selection of good-performing cultivars under sole cropping does not always lead to a good performance in intercropping, the development of an alternative breeding scheme for intercropping is now a necessity. The case study of faba bean–wheat intercropping was used to select for traits associated with better performance of faba bean, resulting in identifying the combined grain yield, 100-seed weight, number of pods per plant, and canopy height as key traits for faba bean–wheat intercropping suitability. Incorporating these traits in the breeding programs would be the cornerstone of the prospective transition.

## 1 Introduction

Agriculture is facing numerous challenges beyond the steep increase of the world’s population, including adaptation to climate change, biodiversity loss, land degradation, and poor agricultural practices. Shifting toward a sustainable and resilient cropping system is therefore needed to avoid the externalities derived from the currently adopted cropping systems. Crop diversification has gained more attention in the last two decades as a potential route toward a sustainable and resilient cropping system (Alletto et al., 2022). As per Hufnagel et al. (2020) definition, crop diversification is “a process that makes a simplified cropping system more diverse in time and space by adding additional crops”. Crop diversification can be implemented through many practices, e.g., cover crops, crop rotation, intercropping, and agroforestry.

Intercropping is one of the sustainable farming system practices that involves growing simultaneously two or more crop species in the same field. It encompasses different aspects such as crop species diversity or different varieties of the same species. There are several types of intercropping systems such as strip intercropping, mixed intercropping, row intercropping, and relay intercropping (Andrews and Kassam, 1976; Bybee-Finley and Ryan, 2018). Intercropping can be presented as a “win–win solution” between farmers and the agroecosystem. Through intercropping, farmers can reduce their income uncertainty by spreading the economic and environmental risks across a wider range of crops, thereby lowering the financial risks brought by poor weather conditions or market shocks (Guvele, 2001). Also, intercropping increases profitability, as it has a demonstrated potential to lower agricultural inputs while improving crop yield and land use efficiency (Himmelstein et al., 2016; Raseduzzaman and Jensen, 2017; Li et al., 2020; Weih et al., 2021).

However, intercropping provides several recognized agroecological benefits like improving the efficient utilization of resources such as soil, water, and radiation (Arina et al., 2021; Xu et al., 2020; Júnior et al., 2023) and enhancing biodiversity (Mala et al., 2020). Moreover, intercropping has demonstrated benefits in alleviating the effects of abiotic stresses on crop productivity (Asgharipour and Rafiei, 2010; Sekar et al., 2019; Yin et al., 2020). Last but not least, intercropping is very advantageous in smothering weeds (Verret et al., 2017), improving the natural suppression of pests (Iverson et al., 2014; Zhang et al., 2019), and enhancing the nutrients uptake of crops leading to a reduction of chemical inputs (Boudreau, 2013; Iverson et al., 2014) and greenhouse gas emissions (Pereira et al., 2022).

The incorporation of legumes in the intercropping system provides ecosystem services and improves the chemical, physical, and biological soil properties. Not to forget that the inclusion of cool-season food legumes (CSFLs) in the intercropping system is of great socio-economic significance, as they are nutrient-dense and are regarded as an inexpensive source of protein-rich food and feed. Adding to that, compared to synthetic fertilizers, legumes provide a cheaper source of N_2_ due to their ability to fix atmospheric nitrogen, and their inclusion in the system will lead to the reduction of their production gap and import in many countries.

Although many studies highlighted the beneficial aspects of intercropping in general and legume-based intercropping in particular (Stagnari et al., 2017; Chamkhi et al., 2022), the acreage of intercropped lands remains low, and intercropping is practiced in countries particularly where water resources are limited in addition to a number of other constraints including non-suitability for mechanization, scarcity of varieties adapted to intercropping, high labor cost, lack of extension and technical support, management complexity, and the demand for a single and standardized product by the market forces, which stimulates the specialization of cropping system. Specific breeding for intercropping is required, particularly for legumes, as they exhibit poor competitive ability (Annicchiarico et al., 2019; Moore et al., 2022, 2023).

In this paper, we start by providing a brief overview of the studies that addressed the incorporation of CSFL crops in intercropping systems. After that, a faba bean (*Vicia faba*)–durum wheat (*Triticum turgidum*) intercropping system will be presented as a case study to give readers a thorough understanding of the traits related to intercropping suitability. Lastly, in order to enhance the integration of CSFLs in the intercropping systems, we propose a breeding strategy and describe the traits to be considered when selecting for intercropping suitability.

## 2 Cool-season food legumes in the intercropping system

While reviewing the intercropping systems that dealt with the inclusion of CSFLs namely, faba bean (*V. faba*), pea (*Pisum sativum*), lentil (*Lens culinaris*), chickpea (*Cicer arietinum*), and grass pea (*Lathyrus sativus*) in the intercropping system, we noticed that the vast majority of studies investigated various combinations of legumes with cereals mainly wheat, barley, and maize (**Table 1**). Among the CSFLs, scientists seemed to have a particular interest in faba bean as an intercrop owing to its shade tolerance (Nasrullahzadeh et al., 2007) and the fact that it fixes more atmospheric nitrogen (N_2_) and has higher protein content than other CSFL including chickpea, field pea, and lentil (Raikos et al., 2014; Chávez-Murillo et al., 2018; Liu et al., 2019). Also, pea appears to have considerable potential as a legume component in the intercropping system (**Table 1**).

**Table 1:** Sample of the research studies conducted on cool season legumes/cereals intercropping.

Legume	Cereal	Location	Purpose	References
Faba bean	Wheat/maize	China	Nitrogen fixation	Fan et al., 2006
Faba bean	Wheat	Ethiopia	Yield, land use efficiency, pest management	Agegnehu et al., 2008
Faba bean	Maize	Iran	Resource use efficiency, best intercropping density	Rezaei-Chianeh et al., 2011
Faba bean, pea	Wheat	France, Italy	Yield	Kammoun et al., 2021; Tavoletti and Merletti, 2022
Faba bean	Wheat	China	Fusarium wilt management	Lv et al., 2020
Faba bean	Wheat	China	Yield, rust	Guo et al., 2021
Faba bean	Barley	Spain	Rust management	Shtaya et al., 2021; Villegas-Fernández et al., 2023
Faba bean/pea	Oat	Spain	*Orobanche crenata* management	Fernández-Aparicio et al., 2007
Faba bean	Barley, oat	Spain, Egypt, Tunisia, Palestine	Chocolate spot management	Fernández-Aparicio et al., 2011
Pea	Barley, triticale	France, Spain, Sweden, Tunisia	Ascochyta blight management	Fernández-Aparicio et al., 2010; Kinane and Lyngkjaer, 2002; Schoeny et al., 2010
Pea	Wheat	Germany	Yield, quality	Timaeus et al., 2022; Kiær et al., 2022
Pea	Barley	Scotland	Quality	Kiær et al., 2022
Pea	Barley	Argentina	Weed management	Poggio, 2005
Pea	Oat	Sweden	Nitrogen fixation	Geijersstam and Mårtensson, 2006
Pea	Maize	China	Yield, water use efficiency	Mao et al., 2012
Lentil	Wheat	India	Growth, yield, quality	Singh et al., 2019
Chickpea	Wheat	France	Nutrient bioavailability	Betencourt et al., 2012
Grass pea	Maize	China	Water and phosphorus availability	Zhu et al., 2022

Regardless of the combinations adopted, the benefits of including CSFL crops in intercropping systems are now well-recognized by researchers and farmers, thanks to the numerous studies conducted over the years (**Table 1**) that thoroughly investigated different aspects of this practice and emphasized its economic return whether it was the result of yield increase or chemical input reduction. Prior studies focused mainly on highlighting and elucidating the beneficial aspects of legumes as an intercrop; evaluating the performance of the intercropping system; determining the best agronomic factors such as sowing density, fertilizer application, row spacing; and evaluating the species compatibility for intercropping regardless of the variety.

Intercropping with CSFL crops offers multiple benefits to the ecosystem. As a matter of fact, legumes enrich the soil with nitrogen due to their ability to fix atmospheric nitrogen (N_2_) as a result of their symbiotic relationship with a soil bacteria called rhizobia, thus boosting the soil organic carbon (SOC) sequestration (Liu et al., 2022). Compared to fossil-based fertilizers, legumes provide a cheaper source of N_2_, thereby lowering the carbon footprint (Bedoussac et al., 2015; Jensen et al., 2020). Another point to consider is that legumes facilitate the phosphorous (P) acquisition by the intercrops due to microbial-mediated processes involving soil fungi and phosphorus-mobilizing bacteria (Mei et al., 2012; Li et al., 2018). In addition, it is important to accentuate that legumes grown in intercropping improve the behavior of the bacteria associated with the roots in the rhizosphere (Chamkhi et al., 2022) and help reduce the soil erosion by providing a denser cover against the striking impact of rainfall on the surface and using their shallow roots to bind the soil particles (Lithourgidis et al., 2011; Dwivedi et al., 2015).

Intercropping CSFLs with other crops increases seed output and improves nutrient yield and seed quality when compared to solely grown crops (Tosti and Guiducci, 2010; Lithourgidis et al., 2011; De Stefanis et al., 2017; Chimonyo et al., 2023; Marcos-Pérez et al., 2023). It is pertinent to mention here that although some scientists reported a small seed yield penalty in intercropping compared to monocropping, they still considered intercropping a beneficial practice, as scientists rely mostly on the land equivalent ratio (LER) as a quantitative measure to assess the performance of the intercropping system and to provide insights into the efficient land utilization in intercropping compared to growing each component crop separately (Mead and Willey, 1980). For instance, Chen et al. (2004) reported lower pea biomass when intercropped with barley compared to when grown separately; however, higher LER was shown in intercropping. Li et al. (2023) reported similar findings later in 2022. Note that an LER value greater than 1 suggests that the intercropping system is more productive than the sole production of crops, which is the case in almost all studies that dealt with the performance of CSFLs in the intercropping system.

Numerous studies have been conducted to better understand the underlying mechanisms behind this yield advantage. Scientists attributed this yield advantage to the low competitiveness and the complementarity use of resources by both intercrops (Agegnehu et al., 2008). For example, an efficient use of light results from the complementary use of space between the taller maize plants and shorter pea plants (Yang et al., 2018). Another explanation for the yield advantage is the more diversified and functional soil microbial communities identified in the legume intercropping system (Tang et al., 2016; Wahbi et al., 2016). Simply stated, legume root exudates contain compounds that enhance the composition and activity of soil microbes. Liu et al. (2020) indicated that faba bean–wheat intercropping positively affected the nitrogen fixation ability of faba bean by increasing the number of nodules in comparison with monocropped faba bean. Also, the findings of Pivato et al. (2021) pointed to a more complex rhizosphere bacterial network in wheat–pea intercropping. The yield increase can also be justified by the facilitation of uptake and utilization of N and P from CSFL crops (Li et al., 2014; Mouradi et al., 2018; Xu et al., 2019). Indeed, in legume intercropping systems, the atmospheric nitrogen fixed by legumes can be used by both intercrops; Li et al. (2021) stated that the nitrogen fixed can be used by cereal in the legume–cereal intercropping system. However, P availability is increased by rhizosphere acidification as a response to nitrogen fixation (Jensen, 1996; Hauggaard-Nielsen et al., 2009), and its mobilization is facilitated by intercropped roots due to different rooting depths (Hauggaard-Nielsen and Jensen, 2005; Betencourt et al., 2012; Latati et al., 2016). In tzhis regard, numerous studies proved the ability of chickpea roots to facilitate the uptake of P by its intercrop companion (Li et al., 2003, 2004).

Several studies have demonstrated that intercropping can significantly reduce the incidence and severity of various diseases. For example, intercropping chickpea with flax suppressed Ascochyta blight, which is a worldwide constraint for chickpea production (Zhou et al., 2023). Similar results reported that intercropping can effectively control foliar and soil-borne diseases in CSFL crops (Schoeny et al., 2010; Mousa and El-Sayed, 2016; Zhang et al., 2019; Lv et al., 2020). The disease reduction is assumed to arise from differences in host physiology, direct pathogen suppression, modified canopy microclimates, decreased host plant density, root exudates, and intercrop barrier effects (Fernández-Aparicio et al., 2010; Schoeny et al., 2010; Boudreau, 2013; Villegas-Fernández et al., 2023). In addition, intercropping has been found to mitigate the effects of harmful weeds. For example, intercropping faba bean or pea with cereals, lupin, fenugreek, Egyptian clover, and garlic markedly reduced the incidence of broomrape, which is a holoparasitic threat for the CSFL production in the Mediterranean area (Bakheit et al., 2002; Fernández-Aparicio et al., 2007; 2008; Abbes et al., 2019; El-Mehy et al., 2022). This reduction in weed incidence is caused by the allelochemicals generated by the intercrop roots that inhibit the germination of weeds, the change in host density, and the alteration in the soil environment (Abbes et al., 2019; El-Mehy et al., 2022).

The proper choice of the variety to be grown in intercropping is crucial because it deeply influences the performance of the whole combination (Hauggaard-Nielsen and Jensen, 2001; Demie et al., 2022; Tavoletti and Merletti, 2022), and even though it has been reported that the performance of a plant grown as a sole crop is poorly correlated to its performance when grown in a mixture (Annicchiarico et al., 2019) and despite the availability of genetic variability for intercropping (Mouradi et al., 2018; El-Mehy et al., 2020; Tavoletti and Merletti, 2022), few studies aimed to select and develop CSFL varieties suitable for intercropping or to at least identify the traits associated with intercropping suitability.

## 3 Intercropping case study: faba bean–durum wheat

This study was conducted with the aim of evaluating the performance of different faba bean breeding lines under different cropping systems and identifying the traits to be considered when selecting faba bean for intercropping suitability. Published data from three different intercropping experiments (Maalouf et al., 2022) were used. These experiments were conducted during the 2019 and 2020 cropping seasons under diverse rainfed conditions at three different research stations of the International Center for Agricultural Research in the Dry Areas (ICARDA): Kafardan and Tal Amara in Lebanon and Marchouch in Morocco. The first two stations have a Mediterranean climate characterized by a wet cold winter and a hot dry summer extending from May to September. The meteorological statements show annual average precipitations of 648 mm and 699 mm. The soil at the Kafardan and Tal Amara stations is deep and has a clay texture. The Marchouch station is characterized by a semi-arid climate with mild winters (intermediate Atlantic rainfed). The meteorological statements show annual average precipitations of 284 mm during the 2019/2020 cropping season. The soil at this experimental station is decalcified vertisol in the upper layer but shows variable carbonation at depth. The soil texture is silty-clayey.

In brief, 40 faba bean breeding lines (**Supplementary Table 1**) with durum wheat variety Margherita were evaluated under three different cropping systems: wheat and faba bean intercropping, sole faba bean, and sole wheat. Additional details about the experiments are described in Maalouf et al. (2021) and Maalouf et al. (2022). Observations on phenological, architectural, and agronomic traits were recorded to assess the effect of intercropping on productivity and crop cycle. The traits days to flowering (DFLR), days to maturity (DMAT), canopy height (CH), canopy reflectance (CR), plant height (PLHT), first pod height (FPH), number of branches per plant (NBP), number of pods per plant (NPP), faba bean grain yield (FBGY), and 100-seed weight (HSW) were recorded for faba bean. For wheat, grain yield (WGY) was recorded. In addition, the combined grain yield (CGY) of faba bean and wheat (FB + W) was calculated.

Combined analysis was conducted using Automatic REML analysis of incomplete-block design modules of GenStat (RRID: SCR_014595) for analysis of variance. Variation among accessions and cropping system was assessed in terms of p-values using the Wald statistic, and the best unbiased phenotypic estimates of accessions were estimated with standard error using best linear unbiased prediction (BLUP) values using GenStat software. BLUP values were used to conduct all downstream analyses.

Correlation analysis was conducted to assess the strength of the relationship between two different faba bean traits under two cropping systems. Principal component analysis (PCA) was also conducted to figure out which traits were influencing the performance of faba bean accessions under different cropping systems. Also, to determine the best faba bean accessions for intercropping, the grain yield LER was assessed for each intercropped faba bean accession following the Bulson et al. (1997) formula:


$$LER=\frac{YI1}{YS1}+\frac{YI2}{YS2}$$

where *YI*1 and *YI*2 are the individual crop yields in intercropping, and *YS*1 and *YS*2 are their yields as sole crops.

Significant differences were observed between breeding lines for all the traits and between plant partners for all studied traits except days to flowering. As for the breeding line × plant partner interaction, significant differences were observed for canopy radiation, number of pods per plant, combined grain yield, and sole grain yield.

Under the two different cropping systems, positive and strong correlations between days to flowering and days to maturity and between plant height and canopy height were observed (**Table 2**). Moreover, under the faba bean sole cropping system, positive and strong correlations were observed between first pod height and canopy height but negative correlation with days to maturity (**Table 2**). However, under faba bean and wheat intercropping system, grain yield showed a positive and strong correlation with canopy height, plant height, 100-seed weight, and number of pods per plant but a negative correlation with days to flowering and maturity (**Table 2**).

**Table 2:** Correlation coefficients between different traits of faba bean under faba bean sole (FB) and faba bean + wheat intercropping (FB + W) systems.

		CGY	CH	CR	DFLR	DMAT	FPH	FBGY	HSW	NBP	NPP
CH	FB	-	-								
	FB + W	−0.22	–								
CR	FB	-	0.24	-							
	FB + W	0.21	−0.10	–							
DFLR	FB	-	−0.53***	−0.18	-						
	FB + W	0.52***	−0.42	0.32	–						
DMAT	FB	-	−0.67***	−0.07	0.92***	-					
	FB + W	0.52***	−0.52***	0.29	0.98***	–					
FPH	FB	-	0.72***	0.10	−0.54***	−0.72***	-				
	FB + W	−0.18	0.42	−0.09	−0.33	−0.32	–				
GY	FB	-	0.15	0.44	0.20	0.24	−0.28	-			
	FB + W	−0.33	0.90***	−0.19	−0.60***	−0.69***	0.22	–			
HSW	FB	-	0.17	0.37	−0.08	−0.14	0.14	0.06	-		
	FB + W	−0.33	0.74***	−0.33	−0.58***	−0.64***	0.13	0.82***	–		
NBP	FB	-	0.09	0.44	−0.15	−0.10	0.12	0.27	0.22	-	
	FB + W	−0.21	0.28	−0.30	−0.03	−0.02	0.02	0.29	0.29	–	
NPP	FB	-	0.05	0.21	−0.10	−0.07	0.17	0.07	−0.33	0.27	-
	FB + W	−0.25	0.77***	−0.16	−0.61***	−0.68***	0.25	0.86***	0.63***	0.30	–
PLHT	FB	-	0.82***	0.45	−0.17	−0.26	0.42	0.41	0.20	0.23	0.09
	FB + W	−0.06	0.92***	−0.05	−0.15	−0.24	0.28	0.75***	0.63***	0.33	0.60***

CGY, combined grain yield; CH, canopy height; CR, canopy reflectance; DFLR, days to flowering; DMAT, days to maturity; FPH, first pod height; FBGY, faba bean grain yield; HSW, 100-seed weight; NBP, number of branches per plant; NPP, number of pods per plant.

*** Significant at the 0.001 probability level.

Under sole faba bean, PCA1 and PCA2 accounted respectively for 99.8% and 0.1% of the total variation (**Table 3**). PCA1 was positively associated with grain yield, while PCA2 was positively associated with canopy height and first pod height and negatively associated with days to flowering. However, under faba bean and wheat mixture, PCA1 and PCA2 accounted respectively for 82.2% and 17.8% of the total variation (**Table 3**). PCA1 was negatively associated with the combined grain yield, while PCA2 was positively associated with grain yield, 100-seed weight, number of pods per plant, and canopy height, which suggest that these traits should be targeted when breeding for intercropping.

**Table 3:** PCA values of traits under faba bean sole (FB) and faba bean + wheat intercropping (FB + W) systems.

Trait	FB		FB + W	
	PCA1	PCA2	PCA1	PCA2
**FBGY**	**0.99**	0.00	0.43	**0.9**
**HSW**	0.06	0.32	0.4	**0.71**
**NBP**	0.27	0.20	0.24	0.21
**NPP**	0.08	0.07	0.33	**0.8**
**CGY**	–	–	**−0.99**	0.1
**CH**	0.15	**0.93**	0.31	**0.85**
**CR**	0.44	0.41	−0.22	−0.11
**DFLR**	0.20	**−0.72**	−0.56	−0.39
**FPH**	−0.28	**0.77**	0.19	0.15
**Percentage of variation**	99.8	0.1	82.2	17.8

FBGY, faba bean grain yield; HSW, 100-seed weight; NBP, number of branches per plant; NPP, number of pods per plant; CGY, combined grain yield; CH, canopy height; CR, canopy reflectance; DFLR, days to flowering; FPH, first pod height; PCA, principal component analysis. Values in Bold represent a high association.

**Figure 1:**
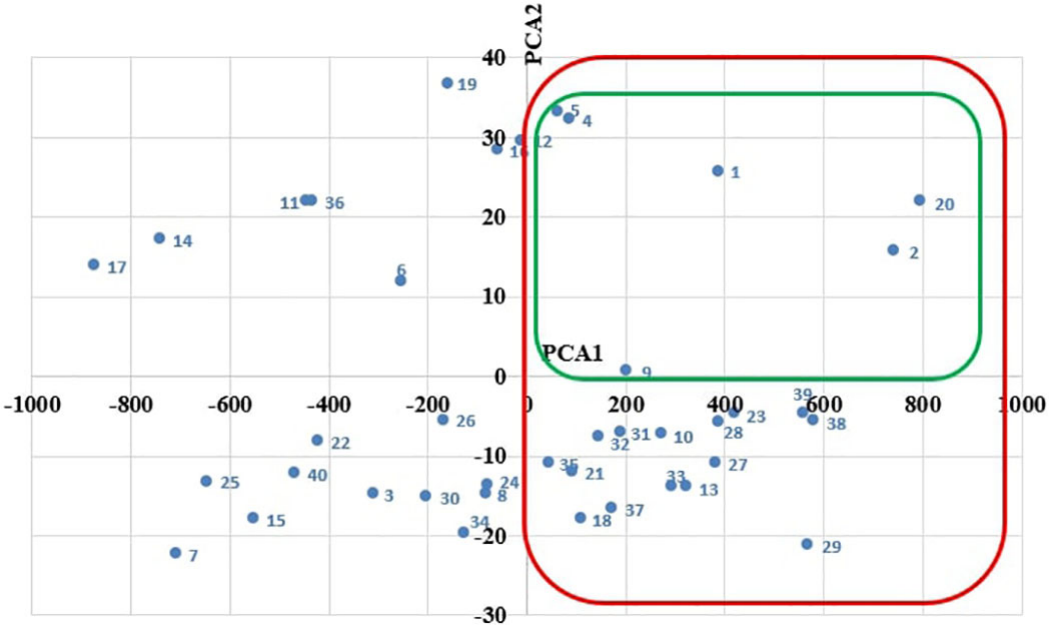
PCA biplot of faba bean under sole cropping system.

The first biplot displays the clustering of the faba bean accessions planted as sole crops based on their performance mainly in terms of grain yield (PCA1) and in terms of canopy height, first pod height, and days to flowering (PCA2) (**Figure 1**). Focusing on PCA1 ordering, **Figure 1** shows that 21 accessions had above-average yield when grown as sole crops (clustered in red), as they lie on the right-hand side of PCA1. Additionally, among these 21 accessions, six (clustered in green) had an above-average canopy height, first pod height, and below-average flowering time, as they were located on the upper side of PCA2 (**Figure 1**).

**Figure 2:**
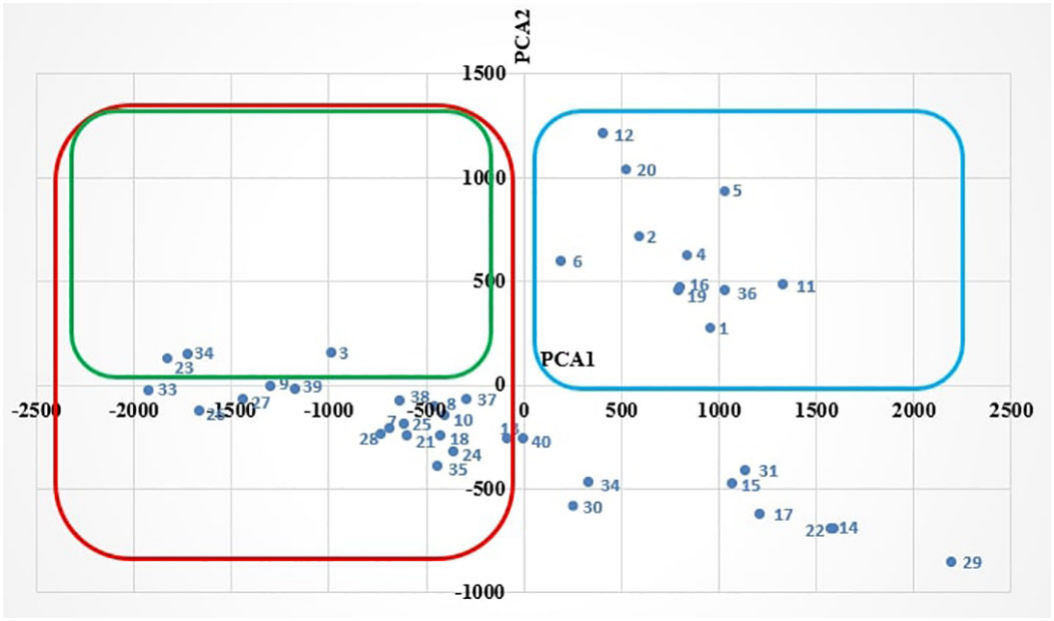
PCA biplot of faba bean intercropped with wheat.

However, the second biplot displays the clustering of the faba bean accessions intercropped with wheat based on their performance mainly in terms of combined grain yield (PCA1), grain yield, 100-seed weight, number of pods per plant, and canopy height (PCA2) (**Figure 2**). Focusing on the PCA1 ordering, **Figure 2** shows that 20 intercropped accessions (clustered in red) had high combined grain yield, as they were located on the left-hand side of PCA1. Also, 14 accessions (clustered in blue) had an above-average faba bean grain yield, 100-seed weight, number of pods per plant, and canopy height, as they were located on the upper side of PCA2. However, the green cluster shows that only three accessions performed well as sole crops and intercrop as measured in terms of grain yield (**Figure 2**). Hence, based on **Figures 1**, **2**, only six accessions showed good performance in both monoculture and intercropping systems.

**Figure 3:**
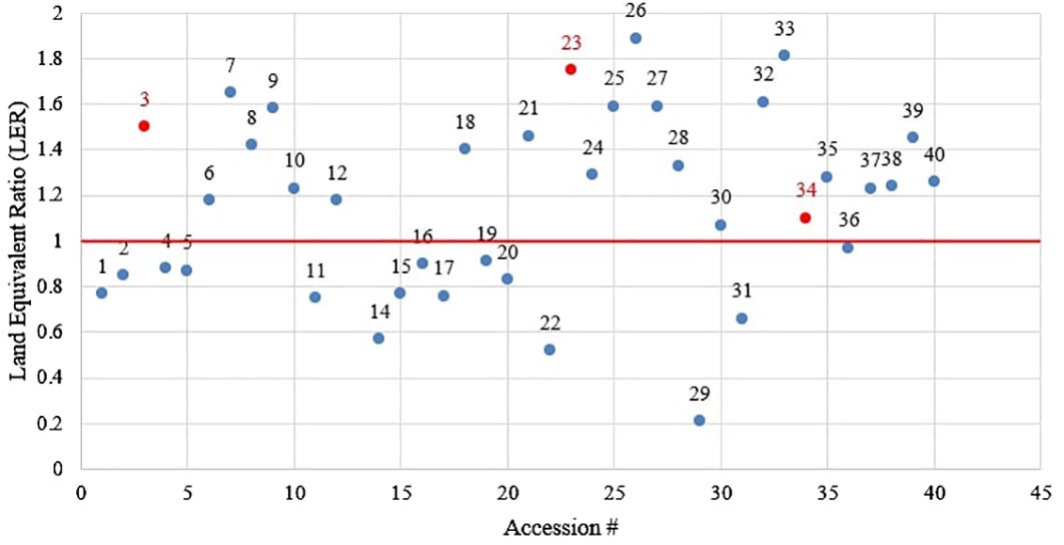
Land Equivalent Ratio of the evaluated faba bean accessions.

The LER confirmed the suitability of these three accessions (numbers 3, 23, and 34) for intercropping with durum wheat. Although some accessions had good LER, their grain yield was not good enough for them to be clustered with good-performing accessions. This might be the result of wheat overyielding, which led to a high combined grain yield, causing an LER above 1. Moreover, the results illustrated in **Figures 2**, **3** show that although some accessions had a good grain yield performance in intercropping (**Figure 2**), they did not perform well in terms of combined grain yield, which was also manifested by an LER value below 1 (**Figure 3**).

## 4 Designing a breeding strategy for intercropping

**Figure 4:**
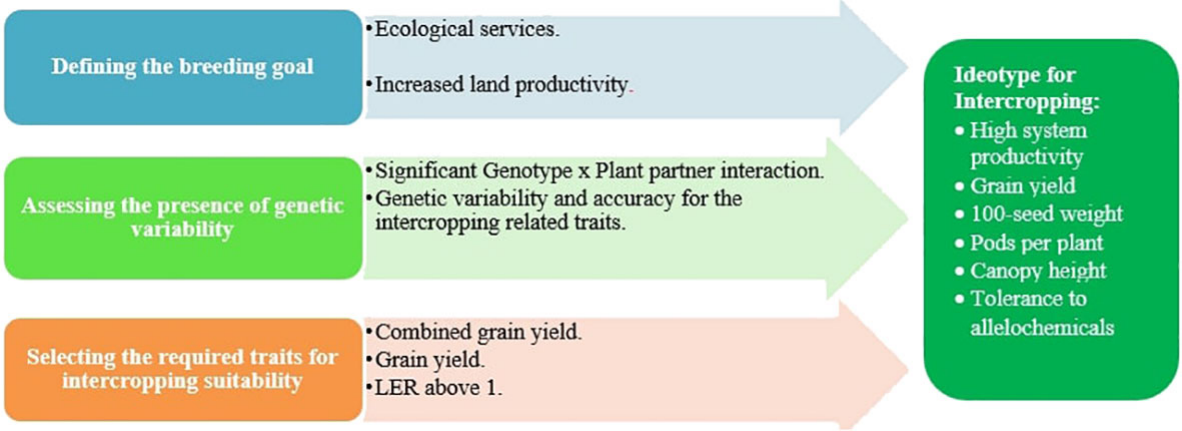
Suggested breeding strategy for intercropping.

As the interest in diversifying the cropping system increases among scientists and farmers, breeding for intercropping suitability has become a necessity. As a matter of fact, before starting any breeding program, breeders must first identify the purpose of the cropping system (**Figure 4**) so that their selection may be based on optimizing the component productivity or system productivity (Moore et al., 2022). Since CSFLs offer an array of benefits as intercrop, many possible purposes can be behind their inclusion in the cropping system. For instance, in a system where CSFLs are planted for ecosystem services, breeders will likely consider them as secondary crops and focus on the yield of the primary crop. However, in a system where they are planted for their nutritional and economic values or in other words as cash crops, breeders will target the total productivity of the system and therefore focus simultaneously on the yield of all intercrops.

Furthermore, breeders must determine if their breeding goals are doable by confirming the presence of a significant genetic variability for intercropping suitability that can be exploited to develop improved cultivars (**Figure 4**). To do so, a comparative study should be conducted by screening different genotypes under different cropping systems (Brooker et al., 2015). Although earlier studies reported the presence of genetic variability in different CSFL crops like faba bean (El-Mehy et al., 2020; Nurgi et al., 2023), pea (Hauggaard-Nielsen and Jensen, 2001; Pankou et al., 2022), and chickpea (Kedar et al., 2000), breeding efforts have been limited. Interestingly, the presented “faba bean–wheat” case study shows a significant breeding line × plant partner interaction, which means that faba bean breeding lines performed differently under different cropping systems. In other words, selecting a good-performing accession in monoculture may not lead to the same results in intercropping, and cultivars intended for monoculture may have different traits than the ones intended for intercropping. Also, the difference observed in faba bean clustering in two different cropping systems highlights the difference in performance under monoculture and intercropping systems, as only 15% of the evaluated accessions had a good grain yield performance in both crop systems. Saxena et al. (2018) reached the same conclusion in their legume–cereal intercropping studies. Although selecting monoculture may be desirable and less complicated, the results obtained emphasized that the need to select for intercropping suitability cannot be made under monoculture. This agrees with the conclusion of Byth et al. (1981) that selection should be made in the environment for which the end product is targeted. Therefore, additional efforts are required to establish an alternative breeding scheme intended for the development of varieties suitable for intercropping.

The key objectives to consider when designing an effective breeding scheme are to discern the target traits and to create an ideotype for intercropping accordingly (**Figure 4**). As per Donald (1968) definition, an ideotype is “a biological model expected to perform in a predictable manner within a defined environment”. This conceptual plant is a combination of morphological and physiological traits and is supposed to have a great yield when developed as a cultivar. Earlier studies have screened traits impacting the performance in intercropping in order to use them in their selection for intercropping suitability and to establish a trait-informed breeding approach (Moore et al., 2022). For example, Short and Carlson (1989) selected intercropping compatibility between orchard grass and bird’s-foot trefoil based on the canopy height, tillering, and maturity, and later on, Maamouri et al. (2017) considered the internode length, shoot number, leaf size, and growth habit as key traits behind the competitive ability of alfalfa. Demie et al. (2022) found that differences in days to maturity, plant height, and growth habit are behind the variations in the performance of the cereal/legume intercropping systems.

In the “faba bean–wheat” case study, results show that breeding for monoculture breeders should focus mainly on faba bean grain yield in addition to the following three traits: canopy height, first pod height, and days to flowering. However, in the case of intercropping, it might be more appropriate to focus mainly on the combined grain yield, 100-seed weight, number of pods per plant, canopy height, and sole grain yield. Although the last two traits influenced the performance of faba bean accessions in both cropping systems, the results obtained confirmed once again that selecting for intercropping based on monoculture data will not be efficient, as the combined grain yield, which happens to be the most influential trait in intercropping, is not observable without intercropping, and therefore, it will be more efficient to adopt the trait-blind approach and select directly in an intercropping system (Barot et al., 2017). Interestingly, results show that under the intercropping system, grain yield was highly correlated with number of pods per plant, plant height, and 100-seed weight, which means that breeders can select for these traits while saving time and resources by only addressing the grain yield in addition to the combined grain yield. Considering this, a good faba bean ideotype for intercropping with wheat is a plant embodying good sole and combined grain yields (**Figure 4**).

As the presented case study is an example of legume–cereal intercropping, the effect of canopy height on the performance of faba bean in intercropping was foreseen, as a higher growth rate is required for legumes to be able to compete against cereals. Barillot et al. (2014) also observed that pea performance under intercropping was affected by leaf area, leaf area development, and plant height. Our results also showed that a larger seed size is required for faba bean to perform well when intercropped with wheat. This feature is of great importance, as a substantial difference in the seed size is recommended between the intercropping partners so that mechanical harvesting and post-harvest separation can be handled successfully. This requirement makes faba bean a great intercrop for wheat due to the difference in their seed size, which is not always the case in other small-seeded legumes like lentils.

Since grain yield appears to influence the performance of faba bean in intercropping, this might be good enough in cases where faba bean is grown as a primary crop. However, since cereals tend to be the crop of interest for the majority of farmers adopting the legume–cereal intercropping system, the yield of both intercrops should be taken into account, and therefore, breeders should focus on the combined grain yield. A good combined grain yield means a positive interaction between the intercrop partners or in other words, niche differentiation, facilitation, and better resource use efficiencies (Lithourgidis et al., 2011; Yu et al., 2015; Xu et al., 2020). This is more likely to happen when functionally different crops are grown together (Verret et al., 2017; Rodriguez et al., 2020) as is the case in the faba bean–wheat case study. In this regard, Hauggaard-Nielsen et al. (2008) reported that beans appear to be more suitable than peas for intercropping with barley cultivars because of a better spatial complementarity between them. In addition, Streit et al. (2019) reported an alteration in the vertical root distribution of intercropped faba bean and wheat allowing better resource utilization, and Xiao et al. (2004) reported that the increase in nutrient uptake efficiency was the main cause behind the yield improvement in intercropped. Hence, as the presented case study involves faba bean, its proven ability to facilitate nutrient acquisition by the intercrop companion can be one of the factors behind high wheat yield. For all these reasons, every suggested intercropping breeding strategy should encompass niche differentiation and facilitation as a way toward an increase in total productivity.

However, a low combined grain yield might be due to the competition between the component crops that leads to one intercrop having an advantage over the other, which reduces the total system productivity (Corre-Hellou et al., 2006; Bybee-Finley et al., 2016). This competitive advantage depends on the traits of the grown variety and its ability to survive under certain growing conditions. For example, the capacity of legumes to fix atmospheric N gives them the ability to survive in conditions of N deficiency more than any other crops. This might explain why in some plots of the studied faba bean–wheat case study we obtained a low combined faba bean and wheat grain yield and a high faba bean grain yield. However, this cannot be confirmed, as the soil composition was not studied.

Also, the low combined grain yield might be due to the allelopathic potential of one of the two intercrops that are negatively impacting the yield of the other intercrop companion. Allelopathy involves the inhibitory effect of a donor plant on both the plant itself and another receiver plant through the release of allelochemicals affecting the establishment and growth of the receiver. The allelopathic potential of different legumes and cereals crops like wheat, sorghum, maize, corn, oats, and soybean has long been studied as an alternative cost-effective tool for the management of insects, diseases, and weeds (Wu et al., 2001; Aslam et al., 2017; Głąb et al., 2017; Dhungana et al., 2019). Also, Makoi and Ndakidemi (2012) reviewed the roles that allelopathy can play as a key player in the protection, defense, and growth stimulation of legume/cereal intercropping systems. Additionally, allelopathy was studied in faba bean–wheat intercropping to reduce the damage caused by faba bean wilt (Guo et al., 2021). Moreover, the allelopathic effect of durum wheat and faba bean against other crops and the risk they present to crop sequences has been reported (Oueslati, 2003; Oueslati et al., 2023). More precisely, Oueslati et al. reported in 2023 that faba bean carries a risk of allelopathic effects when grown as a cover crop preceding durum wheat. However, little attention has been focused on the effect of allelochemicals on the companion crop in intercropping systems. In this context, in their trait selection, breeders may include allelochemical production and susceptibility to allelochemicals as a selection criterion when selecting for intercropping suitability.

Since the performance of a cultivar in intercropping can be modulated by many agronomic factors and given the wide support of the policymakers for the widespread monoculture farming system, breeders alone cannot guarantee a successful adoption of diversified cropping systems like intercropping. Extensive collaboration between many actors including plant breeders, agronomists, farmers, and private and public sectors to develop, promote, and adopt cultivars for intercropping is required.

## Author contributions

LAK: Conceptualization, Visualization, Writing – original draft, Investigation. FM: Conceptualization, Data curation, Formal analysis, Funding acquisition, Investigation, Methodology, Project administration, Resources, Supervision, Writing – review & editing, Validation. RB: Writing – review & editing. YH: Writing – review & editing. XZ: Writing – review & editing. DR: Writing – review & editing. SK: Writing – review & editing.
